# Oil Palm Fruits Ripeness Classification Based on the Characteristics of Protein, Lipid, Carotene, and Guanine/Cytosine from the Raman Spectra

**DOI:** 10.3390/plants11151936

**Published:** 2022-07-26

**Authors:** Gabriel Tan Hong Tzuan, Fazida Hanim Hashim, Thinal Raj, Aqilah Baseri Huddin, Mohd Shaiful Sajab

**Affiliations:** 1 Department of Electrical, Electronic and Systems Engineering, Faculty of Engineering and Built Environment, Universiti Kebangsaan Malaysia, 43600 Bangi, Selangor, Malaysia; gabrieltanhongtzuan@gmail.com (G.T.H.T.); thinal@live.com (T.R.); aqilah@ukm.edu.my (A.B.H.); 2 Research Centre for Sustainable Process Technology (CESPRO), Faculty of Engineering and Built Environment, Universiti Kebangsaan Malaysia, 43600 Bangi, Selangor, Malaysia; mohdshaiful@ukm.edu.my; 3 Department of Chemical and Process Engineering, Faculty of Engineering and Built Environment, Universiti Kebangsaan Malaysia, 43600 Bangi, Selangor, Malaysia

**Keywords:** oil palm, ripeness, chemometrics, ANN, Raman spectrum

## Abstract

The capacity of palm oil production is directly affected by the ripeness of the fresh fruit bunches (FFB) upon harvesting. Conventional harvesting standards rely on rigid harvesting scheduling as well as the number of fruitlets that have loosened from the bunch. Harvesting is usually done every 10 to 14 days, and an FFB is deemed ready to be harvested if there are around 5 to 10 empty sockets on the fruit bunch. Technology aided by imaging techniques relies heavily on the color of the fruit bunch, which is highly dependent on the surrounding light intensities. In this study, Raman spectroscopy is used for ripeness classification of oil palm fruits, based on the molecular assignments extracted from the Raman bands between 1240 cm^−1^ and 1360 cm^−1^. The Raman spectra of 52 oil palm fruit samples which contain the fingerprints of different organic compounds were collected. Signal processing was applied to perform baseline correction and to reduce background noises. Characteristic data of the organic compounds were extracted through deconvolution and curve fitting processes. Subsequently, a correlation study between organic compounds was developed and eight hidden Raman peaks including protein, beta carotene, carotene, lipid, guanine/cytosine, chlorophyll-a, and tryptophan were successfully located. Through ANOVA statistical analysis, a total of six peak intensities from proteins through Amide III (β-sheet), beta-carotene, carotene, lipid, guanine/cytosine, and carotene and one peak location from lipid were found to be significant. An automated oil palm fruit ripeness classification system deployed with artificial neural network (ANN) using the seven signification features showed an overall performance of 97.9% accuracy. An efficient and accurate ripeness classification model which uses seven significant Raman peak features from the correlation analysis between organic compounds was successfully developed.

## 1. Introduction

Oil palm (*Elaeis guineensis*) is an oil producing crop which contributes to almost one third of the world’s vegetable oil [1]. Palm oil is rich in vitamin E (tocopherols and tocotrienols), which is a powerful antioxidant known to help reduce free radicals from the body [2]. Despite its large economic value, sustainable efforts need to be put forth to reduce reliance on land expansion in order to increase productivity.

Oil extraction rate (OER) is used as a measurement tool to evaluate crude palm oil (CPO) production. In addition, OER is also a management tool for evaluating the performance of factories and plantations. This is due to the fact that the profitability of the plantation is greatly influenced by the amount of palm oil produced per hectare. The quality of fresh fruit bunches (FFBs) can be distinguished by observing the ripeness degree of the FFB, in which a ripe FFB should have an OER of at least 21% and free fatty acids (FFA) less than 5% [3]. Currently, the OER and FFA levels of an FFB are determined via chemical analysis [3]. Thus, a non-destructive analysis to determine the OER and FFA levels would be very useful because, in palm oil processing, the quality of fresh oil palm fruit bunches will directly influence the quality of the produced CPO [4]. Physically, the skin color of oil palm fruits will change gradually due to biochemical reactions throughout the ripening process. For example, chlorophyll-a has the highest intensity when the fruit is still unripe and decreases as the fruit ripens. Thus, the ratio of chlorophyll-a to carotenoids can be used as a grading system to identify the ripeness of oil palm fruits or bunches [5].

Traditionally, the ripeness of oil palm fruits is assessed via visual assessment [3]. This is done by counting the number of loose fruits under the tree which indicates the number of fruits that have detached from the bunch [3,6]. This traditional technique, aided by a harvesting schedule every 7 to 10 days or every 10 to 14 days, is still a normal practice for harvesters [3]. This conventional method could lead to losses when the majority of the fruitlets from the bunch are still under-ripe. In addition, loose fruits could have fallen from neighboring trees, gotten stuck in oil palm fronds, or eaten by animals which make the visual ripeness assessment process during harvesting even more difficult [7].

Therefore, a quick and non-destructive method using special instruments to assess the quality of oil palm fruit such as the ripeness level is needed in order to minimize the inaccuracy of FFB grading. There are several alternative methods performed by researchers who use special instruments to detect oil palm fruit ripeness levels [8]. Past researchers have developed automated grading systems which use image processing method to evaluate the red, blue, and green (RBG) elements from the fruit bunch images. Classification methods such as k-Nearest Neighbor (KNN) were also used to compare feature values in each image according to the smallest differences from each study data [9]. However, due to the changes in light intensity throughout the day, images captured at different times may cause some discrepancies when determining the ripeness of oil palm fruits using the computer vision technique [10]. To overcome these problems, this study extends our previous work using Raman spectroscopy for oil palm fruit ripeness classification, due to its high potential in analyzing the biochemical content of a fruit sans light dependency [11,12].

Raman spectroscopy is a non-destructive chemical analysis technique that gives information on the chemical bonds within a molecule. It is based on the knowledge that a high-intensity laser source directed at a molecule will scatter two types of signals; the Rayleigh scattering, which is at the same wavelength of the light source and does not provide much information, and the Raman scattering, which comes off at different wavelengths, gives us information on the chemical structures [13]. A Raman spectrum plots these Raman scattering at different wavelengths, versus the intensity of the scattered light. Each Raman peak on the spectrum represents specific chemical bonding within the sample molecule, for example, C=C and C-H bonding. However, some peaks could be hidden and appear as one peak on the Raman spectrum [14]. Using careful signal processing and feature extraction methods, these molecular assignments could be identified and assigned as significant characteristics of the sample, in this case, oil palm fruits exocarp, and later on, correlated with the ripeness level of the fruit samples [12,15]. This is due to the fact that the Raman spectra can be used as a chemical fingerprint for different organic compounds in which each peak of the Raman spectrum corresponds to an active vibration of the molecule in a particular Raman band [16].

### Background Study

Palm oil is obtained from the processing of oil from the mesocarp of oil palm fruits. The development and maturation of oil palm fruit is a complex biological process beginning with the synthesis of oil followed by the formation of chlorophyll, carotene, tocopherols, and tocotrienols. When the fruit reaches 14 or 15 weeks, chlorophyll in the oil palm fruit will start degrading while carotene starts to form until the fruit fully matures [17]. These biochemical changes in the fruit are made apparent by the change of color in the exocarp (skin). Changes in the exocarp color to bright orange usually indicate high carotene levels. The Raman spectroscopy method has several important features that makes it a valuable technique for analysis in food chemistry as well as space exploration [18]. This method can analyze both organic and inorganic compounds with little or no sample preparation. Moreover, through the Raman spectroscopy clarification method, the determination of the structural composition of compounds becomes easier with in situ analysis capabilities and adaptive capabilities for remote analysis. This method has promising potential in detecting and classifying oil palm fruit ripeness as it can identify compound variations during the fruit ripening process without causing any damage to it [19].

According to Nekvapil, portable Raman spectroscopy is gaining wider applications outside of medical, food sciences, and geological applications due to its convenience, sensitivity, and non-invasive capability for on-site assessment of fruits and vegetables [20]. In addition, portable Raman spectroscopy has been used to monitor plant growth and health, early diagnosis of diseases, and also biotic and abiotic stress on crops [16]. It was also used to conduct analyses on the changes of carotene and chlorophyll intensity on tomato fruits of different ripeness stages [20,21]. Nevertheless, this research emphasizes that, with the existence of unique spectral values, data from the Raman spectra can be extracted and used as important features for machine learning models in developing an automated ripeness classification system.

Machine learning methods using ANN have become increasingly popular in agriculture in recent years. Various classifications for fruit maturity have been carried out for oil palm fruits, bananas, mulberries, tomatoes, and avocadoes. ANN has the ability to perform self-learning using available data and produce good accurate results. In the study of Bensaeed, an ANN model was used to classify oil palm fresh fruit bunches into three classes namely underripe, ripe, and overripe [22]. The accuracy of the ANN classification is determined by the wavelength data, which is directly used as input to the classification algorithm. A set of neurons and the connections between them are the two main components of a network. Unlike mathematical models, the ANN model can learn the relationships between parameters without extracting those relationships, and this has made the ANN a highly functional and valuable tool in classification and modeling. The uniqueness of the application of ANN is having the ability to learn from a given situation through training to improve performance accuracy [23].

The main purpose of this study is to classify the ripeness of oil palm fruitlets using features extracted from the Raman bands between 1240 cm^−1^ and 1360 cm^−1^. The extracted features are fed into an ANN model to automatically classify the fruits into under-ripe, ripe, and over-ripe. This article revisits the methods devised for feature extraction from the Raman spectra to identify important organic compounds throughout the ripening process by Raj et al. [12]. From this study, new characteristics of organic compounds from the Raman spectra were extracted, including chlorophyll-a, proteins (from amide III band, β-sheet), lipids, guanine/cytosine, tryptophan, and carotenes. All molecular assignments were cross-referenced with previous findings from multiple literatures. Significant features were chosen from statistical analysis using ANOVA. Finally, an ANN model was trained to carry out the automated ripeness classification process of oil palm fruits based on the significant features extracted from the Raman spectra.

## 2. Materials and Methods

### 2.1. Oil Palm Fruit Samples

**Figure 1:**
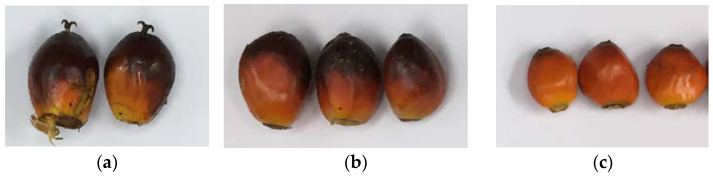
Fresh oil palm fruitlet samples: (**a**) Under-ripe fruitlets; (**b**) Ripe fruitlets; (**c**) Over-ripe fruitlets.

Oil palm fruits samples were collected from the Universiti Kebangsaan, Malaysia (UKM) oil palm plantation (2°54′25.8″ N 101°47′19.8″ E), managed by JANA@UKM (previously known as Khazanah-UKM). The collected samples are of a hybrid species from Elaeis guineensis fo. Dura and Elaeis guineensis fo. Pisifera or better known as Elaeis guineensis DxP whose trees are large and low compared to other species. A total of 52 fruit samples were collected and divided into 2 groups; sample A, which consisted of 5 fruit samples ranging from unripe, under-ripe, ripe, over-ripe, and rotten, and sample B, which consisted of a total of 47 fruit samples, ranging from under-ripe (13 samples), ripe (19 samples) and over-ripe (15 samples). Both group samples were collected a few months apart. The ripeness state of each sample was evaluated by an experienced in-house grader aided by the guideline from the Malaysian Palm Oil Board (MPOB) [24]. Figure 1 shows an example of the color difference between three different maturity stages of oil palm fruits.

### 2.2. Raman Spectroscopy

**Figure 2:**
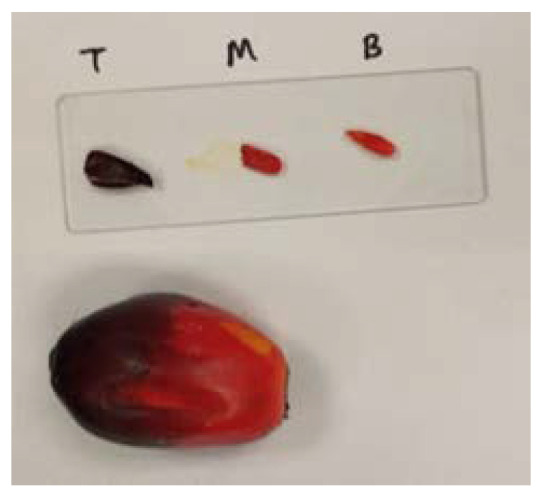
Thin film preparation from the fruit exocarp (T: top, M: middle, B: bottom).

The Raman spectrometer used in this study was the Thermo Scientific, DXR Raman Microscope, set up using a 532 nm laser, green filter, 900 lines/mm grating, and 50 μm slit aperture. Sample preparation could be skipped if there was a fiber optic probe, in which the Raman scattering could be measured directly onto the fruit surface. For this study, a very small and thin portion of the fruit exocarp was peeled and placed on a microscope slide, as depicted in Figure 2. The sample was exposed at 3 different spots (top, middle, and bottom), with a 2.0 mW laser power, for 3 milliseconds. These two parameters were pre-adjusted in the lab to suit the thin film sample and laser wavelength. A too high laser power, for example, could burn the fruit skin. Different laser wavelengths require different exposure times; for example, a longer wavelength such as the 785 nm red laser would require a longer exposure time since the Raman signal will be weak. Since we were using a 532 nm wavelength green laser, which is a shorter wavelength compared to 785 nm, the Raman signal was almost 4 times stronger due to the 1/λ^4^ Rayleigh scattering law. For this reason, an increment in the exposure time could result in more noise in the spectrum. The raw Raman scattering data in the form of Raman shifts (cm^−1^) and intensity (a.u.) were collected in the form of SPA and CSV files. The average spectra from the 3 different spots were used as the modeling spectra.

### 2.3. Raman Spectra Pre-Processing

Next, the spectral data were pre-processed to clean and enhance the spectrum data points. Spectra pre-processing, such as Raman spectra baseline correction, background noise removal, data filtering, and data interpolation, were aimed to modify and rescale the entire data accurately to facilitate the curve fitting process. Spectra baseline correction and data smoothing are common techniques to remove background noise from Raman signals. In this study, the Savitzky-Golay filter was applied to maintain the shape of the Raman peaks so that important aspects from the spectra could be extracted. The retention of the peak curve in Raman spectra was important in this research to accurately identify the molecular characteristics of oil palm fruits. A new value for each data point was created by performing local polynomial regression around each point with the Savitzky-Golay filter. Lastly, linear interpolation was performed to restore lost data at each peak and improve the resolution of the Raman spectra.

#### Signal Deconvolution and Curve Fitting

**Figure 3:**
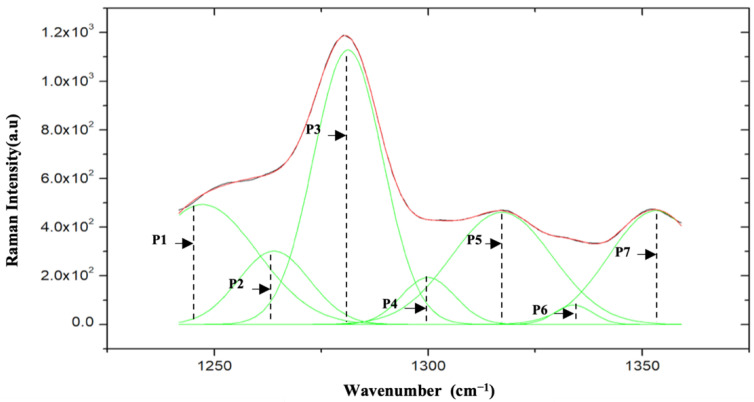
Raman spectra after deconvolution and curve fitting between the range of 1240 cm^−1^ to 1360 cm^−1^, where 7 hidden peaks are revealed, labeled as P1-P7.

After pre-processing, deconvolution was applied to decompose the overlapping peaks and extract information from the hidden peaks [12,14]. All rescaled Raman spectra were resolved into separate component bands in this stage. This technique is based on the original algorithm of nonlinear peak assembly. In this study, OriginPro 2021 was used for spectrum deconvolution where a Gaussian function was applied to allow accurate extraction of molecular features at the peak. Generally, the Lorentzian profile is usually used for decomposing the peaks from Raman spectra [25]. However, several authors have attempted using Gaussian and Voigt profiles, or a mixture of two profiles such as Lorentzian and Gaussian [26]. For this study which focuses on the Raman spectra range from 1240 cm^−1^ to 1360 cm^−1^, we found that the Gaussian profile gave the best fit with the least reduced Chi-squared value. Figure 3 shows the Raman spectra between the range of 1240 cm^−1^ and 1360 cm^−1^ after the deconvolution process. Eight new peaks were identified with seven peaks shown in the figure, labeled P1 to P7 (the eighth peak is very weak and not apparent in the plot), in which these new peaks’ vibrational modes and molecular assignments were identified in Section 3.3.

### 2.4. Correlation Study between Carotene, Chlorophyll-a and other Organic Compounds in the Raman Shifts Range of 1240 cm^−1^ to 1360 cm^−1^

After pre-processing of the Raman spectrum, intensity values from each Raman peaks in between 1240 cm^−1^ and 1360 cm^−1^ were extracted and studied. Different ripeness classes from 52 oil palm fruit samples were analyzed and studied based on findings from previous studies. The peak intensity value of chlorophyll-a decreased throughout the ripening process of the oil palm fruit. At the same time, other organic compounds such as carotene, proteins, and lipids that contribute to the growth and ripening of the fruit rose throughout the process.

### 2.5. Features Extraction of Organic Compounds from the Raman Shifts Range of 1240 cm^−1^ to 1360 cm^−1^

Once each desired peak was successfully fitted with a Gaussian curve, an analytical report which contained the peak features was generated. In addition to the peak intensity, each oil palm fruit sample contained three other main features, such as Gravity Center (peak position), full-width half maximum (FWHM), and Integrated Area. In this study, the ratio between peak features was also calculated. Finally, a total of 27 key features of organic compounds were extracted. Subsequently, the 27 key features of the Raman peaks underwent statistical analysis using ANOVA to determine the most significant features.

### 2.6. Development of Classification System Based on Artificial Neural Network

An ANN classifier was developed in MATLAB by Mathworks to classify different ripeness classes of the oil palm fruits. In this study, an ANN classification model based on a fully connected multilayer perceptron (MLP) with backpropagation was built in which the input layers for the model were fed with 7 significant features extracted from ANOVA analysis. These significant features were the peak intensities from proteins through Amide III (β-sheet), beta-carotene, carotene, lipid, guanine/cytosine, and carotene, and the peak position of lipid. Next, the hidden layer was set to 20 neurons to analyze deeper features of the input. We found that this setup led us to achieve the best percentage in accuracy in a short computational time. The output layer was formed by three neurons representing the under-ripe, ripe, and over-ripe classes. The data set was divided into training, validation, and testing sets in the ratio of 60% to 10% to 30% respectively. A total of 33 samples from sample B were used to train and validate the model and 14 samples were used as a test dataset. Although 47 seems like a small dataset, it is reported that the limit on accuracy for an ANN model is determined by the noise in the dataset and was known to perform well for datasets less than 50 [27]. ANN models are usually suitable for problems with enough data or observation. However, the amount of data for training is also dependent on the network structure, the training method, the complexity of the problem, and the amount of noise in the dataset [27].

## 3. Results and Discussion

### 3.1. Identification of Chlorophyll-a Vibration Mode in the Raman Spectra of Oil Palm Fruits without Spectrum Pre-Processing

**Figure 4:**
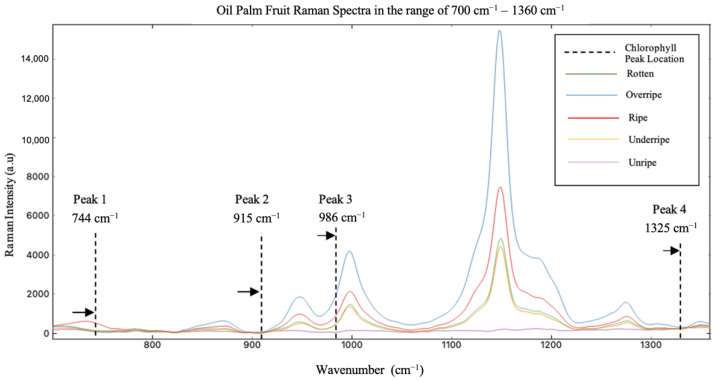
Raman spectra of oil palm fruit in the range from 700 to 1360 cm^−1^.

Figure 4 shows the Raman spectra obtained from five different fresh oil palm fruitlets from sample A. A total of four Raman peak positions ranging from 700 cm^−1^ to 1360 cm^−1^ were identified as chlorophyll-a according to their vibrational modes and molecular assignments observed by previous studies, as summarized in Table 1. Trebolazabala et al. applied the Raman spectroscopy method to monitor the ripening process of tomatoes by studying their skin characteristics. The study revealed that the Raman bands existing at positions between 742–744 cm^−1^, 915 cm^−1^, 982–985 cm^−1^, and 1325 cm^−1^ were derived from chlorophyll-a compounds [19,21]. In this study, four chlorophyll-a vibrational modes were identified in the Raman spectra of oil palm fruit at positions 744 cm^−1^, 915 cm^−1^, 986 cm^−1^, and 1325 cm^−1^, without performing pre-processing on the spectra. The first peak was found to be from the vibration of N-C-C molecules contained in the chlorophyll-a pigment. Moreover, the second peak at position 914 cm^−1^ was found to result from the vibration of the C-C-C molecule, while the third peak at position 986 cm^−1^ was found to result from the vibration of the CH_3_ molecule. Finally, the fourth peak at 1325 cm^−1^ was found to result from the vibration of the CH molecule. The Raman peak position reported in this study was found to be consistent with at least two other studies, Heraud and Jehlicka [28,29].

### 3.2. Features Comparison between the Raman Spectra of Oil Palm Fruits without Spectrum Pre-Processing

In this section, a comparison between two different samples, sample A and sample B, of the features extracted from the Raman spectra and ripeness level was carried out without performing pre-processing on the spectra.

#### 3.2.1. Sample A

**Figure 5:**
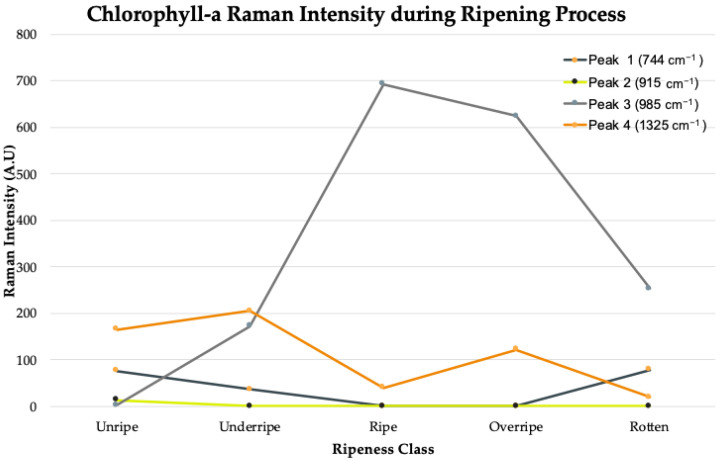
Trend sample A for Raman peak intensity value of chlorophyll-a in fresh oil palm fruit throughout ripening process.

Figure 5 shows the trend for the Raman peak intensity for chlorophyll-a throughout the oil palm fruit ripening process. The positions of each peak were found at wavenumbers 744 cm^−1^, 915 cm^−1^, 985 cm^−1^, and 1325 cm^−1^. The Raman intensity value of chlorophyll-a should decrease throughout the ripening process. However, the intensity value at the first peak decreased when the fruit turned from unripe to under-ripe and rose back when the fruit went into rotten from the over-ripe class. Moreover, the intensity value at the second peak could not be found. This is suspected to be due to the weak chlorophyll-a peak obtained from the green laser source with 532 nm excitation that caused interference among chlorophyll-a at peak 2 with other organic compounds. The intensity values for the third peak resembled background noise and overlapping signals. Finally, the fourth peak showed an upward trend of chlorophyll-a from under-ripe and over-ripe and a decreasing trend from ripe to rotten.

The relationship between the position of the Raman peaks and the molecular assignments of the organic compounds is summarized in Table 2. As a conclusion, the chlorophyll-a intensity values at each stage of the ripening process for sample A showed a varying trend and were inconsistent with scientific knowledge, that the intensity of chlorophyll-a should decrease throughout the ripening process. Chlorophyllase is an enzyme that catalyzes chlorophyll by hydrolyzed phytol groups, resulting in the formation of chlorophyllides. These enzymes are found in chloroplasts, and these organelles undergo a decrease before and during ripening which is shown in the discoloration of the fruit skin [30].

#### 3.2.2. Sample B

**Figure 6:**
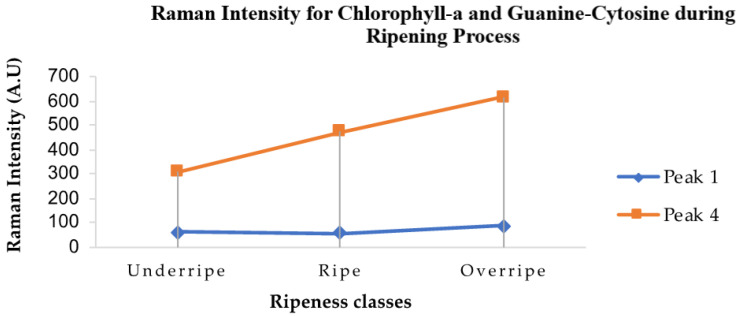
Trend sample B for Raman peak intensity value of chlorophyll-a and guanine-cytosine in fresh oil palm fruit throughout ripening process.

Figure 6 shows the trend for the Raman peak intensity for chlorophyll-a in fresh oil palm fruit ripening process for sample B. The intensity values for the second and third peaks could not be determined. Without spectrum pre-processing, since sample B was bigger than sample A, the first peak position was found at the wavenumber between 744 cm^−1^ and 746 cm^−1^, while the fourth peak position was found between 1317 cm^−1^ and 1318 cm^−1^. In addition, molecules of other organic compounds were also observed in this sample. The peak position of chlorophyll-a in this sample however underwent a shift from 1325 cm^−1^ to a wavenumber ranging from 1317 cm^−1^ to 1318 cm^−1^.

The relationship between the position of the Raman peaks and the molecular assignments of the organic compounds is summarized in Table 3. In this sample, the fourth peak with wavenumber between 1317 cm^−1^ and 1318 cm^−1^ was found to be consistent with guanine/cytosine which are nucleic acids originating from in-plane vibrations of the nucleic acid base [31]. The guanine/cytosine content of the *E. guineensis* genome is said to be around 37% which is similar to other plant genomes [32]. Furthermore, according to a previous study, nucleosides such as guanosine and cytidine were found to be significantly higher in high-yield oil palm fruits during the mature stages of fruit development [33]. The Raman intensities of the second and third peaks were found to be the least significant. This is due to the existence of Raman bands correlated with other vibration molecules contained in the sample such as carotenoid. Therefore, the Raman band containing 1317 cm^−1^ to 1325 cm^−1^ was of interest to be decomposed in order to locate hidden peaks.

### 3.3. Findings from Correlation Study between Chlorophyll-a, Carotene, and other Organic Compounds in the Range of 1240 cm^−1^ to 1360 cm^−1^ after Spectrum Pre-Processing and Deconvolution

In this section, spectrum pre-processing and deconvolution were performed on sample A and sample B for the wavenumber of interest, between 1240 cm^−1^ and 1360 cm^−1^ to locate hidden peaks. After data interpolation, a Gaussian function was applied to recover the original Raman band. The Gaussian curve was successfully fixed at eight peaks in between the range of 1240 cm^−1^ to 1360 cm^−1^. A total of four ripeness classes which were unripe, under-ripe, ripe, and over-ripe from sample A (rotten was excluded due to irrelevancy), and a total of three ripeness classes, which were under-ripe, ripe, and over-ripe from sample B were analyzed after the deconvolution process.

A total of eight Raman peaks labeled P1, P2, P3, P4, P5, P6, P7, and P8 were identified after the process of deconvolution and curve fitting. All eight peaks had different positions and molecular vibrations. The Raman bands and their molecular assignments and intensity values are summarized in Table 4 and Table 5 for sample A and sample B, respectively. The peaks from the Raman bands were found to be derived from the vibrational modes of various organic compounds, such as chlorophyll-a, proteins, lipids, guanine/cytosine, tryptophan, and carotenes.

As reported in previous literature, the first Raman peak (P1) positioned around 1244 cm^−1^ is associated with a protein molecule through the amide III band (β-sheet) [34,35,36]. The second Raman peak (P2) which was positioned next to the first peak at 1258 cm^−1^ is found to be associated with beta carotene molecule [18,24]. The third peak (P3) and eighth peak (P8) at 1281 cm^−1^ and 1357 cm^−1^ were found to be assigned to the same organic compound which is carotene [19,21,29]. The fourth peak (P4) positioned around 1306 cm^−1^ is identified as a lipid molecule which is closest to the peak position found in the literature at 1302 cm^−1^ [19,21]. The fifth Raman band (P5) having a peak position value at 1318 cm^−1^ which is close to the sixth peak was found to be associated with guanine/cytosine. This finding was cross-referenced with the molecular assignments for guanine and cytosine found by Hildebrandt [31]. The sixth peak (P6) at 1325 cm^−1^ that showed a downward trend was reported by Trebolazabala, Heraud, and Jehlička as chlorophyll-a molecules [19,21,28,29]. Finally, the seventh peak (P7) positioned around 1335 cm^−1^ can be associated with the tryptophan molecule [34,37]. Tryptophan biosynthesis plays a direct role in regulating plant development, response to pathogen defenses, and subsequently insect–plant interactions [38]. Interestingly, Tryptophan (Trp) was the only peak that showed an upward and downward trend throughout the ripening process for both samples, with the highest peak intensity at the ripe stage. This trend is consistent with the findings of the, where Trp is the only amino acid in oil palm fruits that does not show a declining trend throughout the ripening process from 12 to 22 weeks after pollination (WAP) like any other amino acids concentrations observed [33]. It is astounding that the upward and downward trend of Trp found in this study was exactly similar to the findings of Teh in [33].

**Figure 7:**
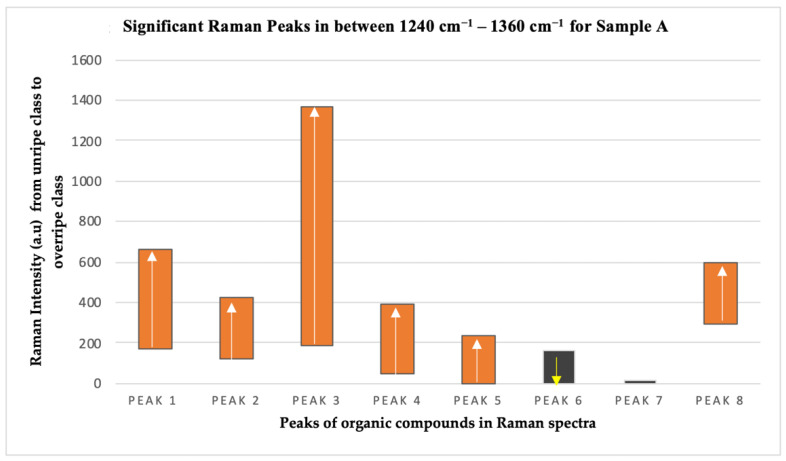
Trend of Raman peak intensity for the four ripeness classes in sample A.

**Figure 8:**
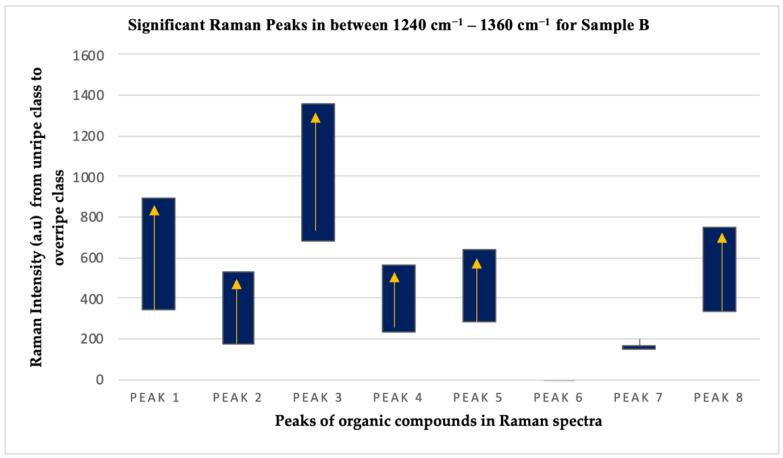
Trend of Raman peak intensity for the three ripeness classes in sample B.

The Raman intensity values of P1, P2, P3, P4, P5, and P8 peaks showed similar trends except for P6 and P7 peaks, as depicted in Figure 7 and Figure 8, for both sample A and sample B, respectively. This means that the organic compounds contained in the exocarp of the oil palm fruits from both samples show a consistent trend throughout the ripening process. The Raman intensity values for the six peaks consisting of proteins, beta carotene, carotene, lipid, and guanine/cytosine increased throughout the ripening process for both sets of samples, while the Raman intensity values for chlorophyll-a from sample A showed a decreasing trend.

In conclusion, new molecular assignments were found after performing deconvolution between the Raman bands of 1240 cm^−1^ to 1360 cm^−1^ and were assigned to chlorophyll-a, proteins (from amide III band, β-sheet), lipids, guanine/cytosine, tryptophan, and carotenes which were cross-referenced with previous literature. The Raman intensities of protein, beta carotene, carotene, lipids, and guanine/cytosine for sample A increased while the Raman intensity of chlorophyll-a decreased throughout the ripening process. According to the study by Trebolazabala, chlorophyll-a content decreases when carotenoid content increases throughout the tomato fruit ripening process [19]. The chlorophyll-a content can also be observed to fade slowly from unripe to ripe fruit. The intensity value for chlorophyll-a was the highest in the unripe class. For sample B, the chlorophyll-a peak could not be determined for all 47 samples. This is likely due to the interference of organic compounds close to the chlorophyll-a peak or a weak Raman signal caused by the 532 nm green laser. It is important to note that some vibrational modes were more apparent when using a 785 nm red laser and some were more apparent when using a 532 nm green laser [21].

### 3.4. Extraction of Significant Features from Raman Spectra between 1240 cm^−1^–1360 cm^−1^

This section presents the results of the features extraction from sample B after the deconvolution and the curve fitting process. Sample B consisted of a total of 47 fruit samples and was used as the dataset for the ANN model. From the correlation results in Section 3.3, a total of seven Raman peaks (excluding P6) are known to be reliable and valid after cross-referencing with past research. A total of 27 features related to Raman peak properties were extracted. Among the peak characteristics extracted were FWHM, peak intensity, peak position, and intensity ratio. After ANOVA statistical analysis test, a total of seven significant features where six from the Raman peak intensities and one from the Raman peak position were identified. The significant features were the peak intensities from P1, P2, P3, P4, P5, and P8, and the peak position of P4. These significant features came from the molecular assignments of proteins from amide III, beta carotene, carotene, lipid, and guanine/cytosine. These seven significant features were used in the development of a ripeness classification system based on the ANN model.

### 3.5. Development of Oil Palm Fruit Ripeness Classification System Based on Artificial Neural Network

**Figure 9:**
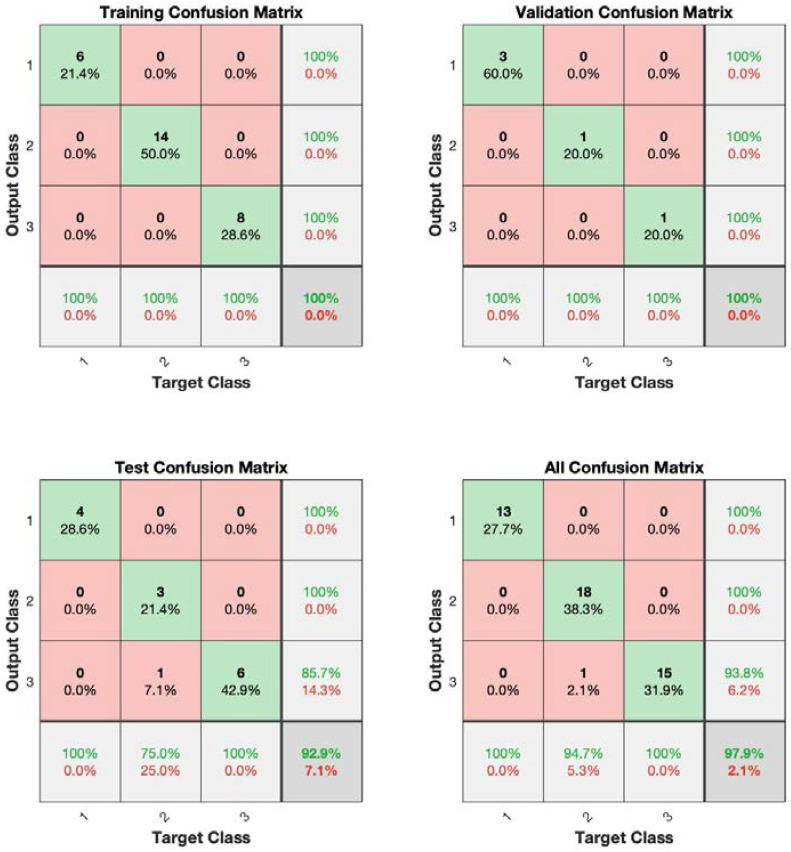
All Confusion Matrix generated from ANN model based on seven significant features.

In this study, a ripeness classification system was constructed to determine the ripeness of oil palm fruits based on the ANN model. This network layer consisted of three parts which were the input layer, hidden layer, and output layer. The input layer consisted of neurons that received input data from significant features of the Raman peak. In addition, a hidden layer consisting of 20 neurons receiving data from the input layer was used to extract deeper features. Finally, the output layer of this network consisted of three neurons to represent the classes of the oil palm fruit maturity. In this model, the cross-entropy error was maximum at the beginning of the training and decreased until reaching the best validation performance at epoch 10. Moreover, the value of cross entropy error converged after 10 epochs with a value that was close to zero which was 0.040468. Figure 9 shows the overall confusion matrix which was a combination of the training confusion matrix, the validation confusion matrix, and the testing confusion matrix. For this classification system which used seven significant features from the Raman peaks as inputs, it managed to achieve an overall performance of 97.9% with only one sample mismatched. Table 6 shows the performance of previous works using different techniques paired with machine learning. It can be seen that the work of Bensaeed in 2014 is the only non-Raman approach with a higher-performing method, due to its usage of hyperspectral images. Hyperspectral images however are known to have limitations of being heavy in storage and high in computational cost which could be a limitation for a rapid and portable solution in oil palm plantations. The study by Raj et al. in 2021 achieved higher accuracy using Raman spectroscopy and KNN due to the extraction of features between the 1495 cm^−1^ to 1535 cm^−1^ band which consisted of stronger molecular assignments of beta carotene, lutein, lycopene, and neoxanthin [12].

### 3.6. General Comments

This study explored the Raman band between 1240 cm^−1^ and 1360 cm^−1^ to look for important molecular assignments based on the vibrational modes of the molecules in the oil palm fruit exocarp. This study made use of chemometrics, from a combination of both signal processing and biochemical approach in order to generate a more accurate classification model to classify the ripeness of oil palm fruits.

In the early stage of this study when the Raman spectra from Sample A did not undergo the signal pre-processing process, the signal contained high frequency and background noise before deconvolution and curve fitting was performed. This acted as a blinded test to compare the results of this study obtained from the raw signal with the results from previous researchers. The peaks forming the Raman spectra were formed from various vibrational modes of the molecules which could be assigned to specific molecular assignments. The deconvolution process needed to be implemented to restore the hidden peaks forming the Raman spectra.

Chlorophyll-a intensity found in Section 3.2.1 was very inconsistent due to the absence of spectrum filtering and interpolation. In addition, the 532 nm green laser is known to be weak in observing chlorophylls but is excellent for observing other Raman bands such as cuticular compounds [21]. In conclusion, 785 nm red lasers are more suitable for observing strong chlorophyll-a pigments and green lasers are more suitable for observing carotene pigments. The non-invasive detection method also allowed organic compounds contained in the exocarp of fresh oil palm fruit to be identified without damaging the fruit.

## 4. Conclusions

An oil palm ripeness classification model based on the properties of carotene and other organic compounds found between 1240 cm^−1^ and 1360 cm^−1^ Raman band was developed in this study. A total of seven organic compounds, including protein, beta carotene, carotene, lipids, guanine/cytosine, chlorophyll-a, and tryptophan were successfully extracted through Raman spectrum pre-processing, deconvolution, and curve fitting within this Raman band. The existence and presence of organic compounds found in the oil palm fruit exocarp was confirmed through the increment and decrement trends of the Raman peak intensities obtained through Raman spectroscopy. The molecular assignments of the organic compounds found in this study were cross-referenced with other literature. Thus, it was proven that Raman spectroscopy can provide important and useful information that can be used for fruit quality assessment. A total of 27 key features from the organic compounds were extracted from the FWHM, peak intensity, peak position, and intensity ratio. From this, seven significant features were fed into an ANN model to classify the datasets collected from UKM oil palm plantation into under-ripe, ripe, and over-ripe categories. The overall performance of the oil palm fruit ripeness classification system showed a 97.9% accuracy. This method which incorporates machine learning and Raman spectroscopy technique has the potential to be a rapid and portable solution for ripeness assessment at oil palm plantations.

## Figures and Tables

**Table 1:** Summary of Raman bands and their molecular assignments and vibrational modes from this study and previous works.

Peak	This Study (cm^−1^)	Villar et al. 2005 [18] (cm^−1^)	Heraud et al. 2007 [28] (cm^−1^)	Jehlicka et al. 2014 [29] (cm^−1^)	Trebolazabala et al. 2017 [19,21] (cm^−1^)	Molecular Assignment	Vibrational Mode
Peak 1	744	n/a	744	757	742–744	Chlorophyll-a	δ(N−C−C)
Peak 2	915	n/a	915	900–915	915	Chlorophyll-a	δ(C−C−C)
Peak 3	986	n/a	988	986	982–985	Chlorophyll-a	*δ(CH_3_)*
Peak 4	1325	1340	1325	1325	1325	Chlorophyll-a	δ(CH)

**Table 2:** Summary of Raman bands and their molecular assignments with intensity values for five oil palm fruit ripening classes for sample A without spectrum pre-processing.

Peak	Band (Raman Peak cm^−1^)	Molecular Assignment	Chlorophyll-a Raman Peak Intensity Value (a.u)				
			Unripe	Under Ripe	Ripe	Over Ripe	Rotten
Peak 1	744	Chlorophyll-a [19,21,28,29]	76.37	36.06	-	-	78.73
Peak 2	915	Chlorophyll-a [19,21,28,29]	13.35	-	-	-	-
Peak 3	985	Chlorophyll-a [19,21,28,29]	2.00	172.70	692.75	623.58	253.20
Peak 4	1325	Chlorophyll-a [18,19,21,28,29]	164.65	204.40	39.33	122.13	18.69

**Table 3:** Summary of Raman bands and their molecular assignments with intensity values for three oil palm fruit ripening classes for sample B without spectrum pre-processing.

Peak	Band (Raman Peak cm^−1^)	Molecular Assignment	Organic Compounds Raman Peak Intensity Value (a.u)		
			Under Ripe	Ripe	Over Ripe
Peak 1	744–746	Chlorophyll-a [19,21,28,29]	61.01	57.17	84.79
Peak 2	915	Chlorophyll-a [19,21,28,29]	-	-	-
Peak 3	986	Chlorophyll-a [19,21,28,29]	-	-	-
Peak 4	1317–1318	Guanine-Cytosine [31]	306.03	472.78	616.37

**Table 4:** Summary of Raman bands and their molecular assignments with intensity values for four oil palm fruit ripening classes for sample A in the range of 1240 cm^−1^ to 1360 cm^−1^ after spectrum pre-processing and deconvolution.

Peak	Band (Raman Peak cm^−1^)	Molecular Assignment	Organic Compounds Raman Peak Intensity Value (a.u)			
			Unripe	Under Ripe	Ripe	Over Ripe
P1	1244	Proteins through Amide III (β-sheet) [34,35,36]	172.79	205.08	385.40	665.57
P2	1258	Beta carotene [18,24]	118.42	127.57	184.36	424.79
P3	1281	Carotene [18]	185.73	413.50	778.01	1364.08
P4	1306	Lipid [18,20]	45.78	200.61	240.54	391.19
P5	1318	Guanine/cytosine [29]	-	13.43	43.44	232.21
P6	1325	Chlorophyll-a [18,23,24]	161.66	35.88	26.40	-
P7	1335	Tryptophan [32,33]	16.23	-	-	-
P8	1357	Carotene [18,20]	293.68318	361.46494	421.66796	597.97796

**Table 5:** Summary of Raman bands and their molecular assignments with intensity values for three oil palm fruit ripening classes for sample B in the range of 1240 cm^−1^ to 1360 cm^−1^ after spectrum pre-processing and deconvolution.

Peak	Band (Raman Peak cm^−1^)	Molecular Assignment	Organic Compounds Raman Peak Intensity Value (a.u)		
			Under Ripe	Ripe	Over Ripe
P1	1244–1250	Proteins through Amide III (β-sheet) [34,35,36]	343.00	701.73	894.58
P2	1261–1266	Beta carotene [18,24]	174.87	357.81	526.63
P3	1280–1281	Carotene [18]	685.53	1134.91	1354.20
P4	1297–1305	Lipid [18,20]	233.06	448.38	563.21
P5	1317–1320	Guanine-cytosine [29]	283.94	411.87	637.68
P6	1325	Chlorophyll-a [18,23,24]	-	-	-
P7	1331–1335	Tryptophan [32,33]	147.96	205.07	169.18
P8	1351–1354	Carotene [18,20]	333.58	568.25	749.752

**Table 6:** Classification results from previous researchers and this study.

Researcher	Overall Performance	Algorithm	Technique
(Shabdin et al. 2016) [39]	70%	ANN	HIS color model
(Bensaeed et al. 2014) [22]	98.67%	ANN	Hyperspectral
(May et al. 2011) [40]	88.74%	Fuzzy logic	RGB color model
(Sameen et al. 2015) [41]	67.10%	Genetic Algorithm	Image processing
(Astuti et al. 2019) [9]	65%	KNN	Sobel edge detection
(Raj et al. 2021) [12]	100%	KNN	Raman spectroscopy (1495 to 1535 cm^−1^ band)
This study (2022)	97.9%	ANN	Raman spectroscopy (1240 to 1360 cm^−1^)

## Data Availability

All oil palm fruit samples were collected with consent from Universiti Kebangsaan Malaysia’s oil palm plantation, managed by JANA@UKM (previously known as Khazanah-UKM).
